# Using a Clustering Approach to Investigate Socio-Environmental Inequality in Preterm Birth—A Study Conducted at Fine Spatial Scale in Paris (France)

**DOI:** 10.3390/ijerph15091895

**Published:** 2018-08-31

**Authors:** Severine Deguen, Nina Ahlers, Morgane Gilles, Arlette Danzon, Marion Carayol, Denis Zmirou-Navier, Wahida Kihal-Talantikite

**Affiliations:** 1 School of Public Health (EHESP), DSET&GS, 35043 Rennes CEDEX, France; Nina.Ahlers@ucsf.edu (N.A.); morgane.gilles@eleve.ensai.fr (M.G.); denis.zmirou@ehesp.fr (D.Z.-N.); 2 Department of Social Epidemiology, Institut Pierre Louis d’Epidémiologie et de Santé Publique (UMRS 1136), Sorbonne Universités, UPMC Univ Paris 06, INSERM, 75012 Paris, France; 3 Service de Protection Maternelle et Infantile, Direction des Familles et de la Petite Enfance, Mairie de Paris, 75196 Paris, France; arlette.danzon@paris.fr (A.D.); marion.carayol@paris.fr (M.C.); 4 School of medicine, Lorraine University, 54000 Nancy, France; 5 Inserm, Irset (Institut de recherche en santé, environnement et travail)—UMR-S 1085, F-35000 Rennes, France; 6 Laboratoire Image Ville Environnement, LIVE UMR 7362 CNRS, University of Strasbourg, 6700 Strasbourg, France; wahida.kihal@live-cnrs.unistra.fr

**Keywords:** air pollution, neighborhood deprivation index, preterm birth, spatial approach

## Abstract

*Background & Objectives*: Today, to support public policies aiming to tackle environmental and health inequality, identification and monitoring of the spatial pattern of adverse birth outcomes are crucial. Spatial identification of the more vulnerable population to air pollution may orient health interventions. In this context, the objective of this study is to investigate the geographical distribution of the risk of preterm birth (PTB, gestational age ≤36 weeks) at the census block level in in city of Paris, France. We also aimed to assess the implication of neighborhood characteristics including air pollution and socio-economic deprivation. *Material & Methods*: Newborn health data are available from the first birth certificate registered by the Maternal and Child Care department of Paris. All PTB from January 2008 to December 2011 were geocoded at the mother residential census block. Each census block was assigned a socioeconomic deprivation level and annual average ambient concentrations of NO_2_. A spatial clustering approach was used to investigate the spatial distribution of PTB. *Results*: Our results highlight that PTB is non-randomly spatially distributed, with a cluster of high risk in the northeastern area of Paris (RR = 1.15; *p* = 0.06). After adjustment for socio-economic deprivation and NO_2_ concentrations, this cluster becomes not statistically significant or shifts suggesting that these characteristics explain the spatial distribution of PTB; further, their combination shows an interaction in comparison with SES or NO_2_ levels alone. *Conclusions*: Our results may inform the decision makers about the areas where public health efforts should be strengthened to tackle the risk of PTB and to choose the most appropriate and specific community-oriented health interventions.

## 1. Introduction

Adverse birth outcomes are important public health issues including preterm birth (PTB) and low birthweight (LBW). Over the past 20 years, the literature confirmed that, in developed countries, PTB remains a risk factor of adverse health outcomes including neonatal mortality and short- and long-term morbidity [1,2,3,4,5,6,7,8]. PTB is also recognized as a risk factor for LBW, delayed motor and social skills, as well as learning disabilities [9]. 

Various contextual determinants, characterizing place where people live, have been reported to be associated with births outcomes, including socio-economic, demographic characteristics and environmental factors such as exposure to environmental contaminants. Several studies concluded that prenatal development is a window of high susceptibility to the adverse impact of environmental nuisances, in particular ambient air pollution [10,11,12,13,14,15,16,17] More specifically, studies revealed that maternal air pollution exposure such as particulate matter ≤10 and ≤2.5 μm in diameter (PM_10_ and PM_2.5_) and nitrogen dioxide (NO_2_) reduced birth weight and increased the odds of low birth weight and preterm birth, [11,12,18]. While findings for PTB remains inconsistent in the literature (depending, in particular, on the study design, exposure assessment, pregnancy periods and adjustment for confounders [12,18,19,20,21]), experimental studies support plausible biological mechanisms explaining, for instance, how air pollution exposure could reduce gestational age via placental inflammation linked to oxidative stress [22].

In addition, some studies suggested that the adverse health effect of maternal environmental exposure may be influenced by other contextual or individual characteristics (such as sex, socioeconomic position and psychological factors [12,23,24,25]). Many authors concluded that health risk related to environmental exposure may be different according to the socioeconomic level of populations [26,27,28,29,30]. For instance, Yi et al. in 2010 found a three-fold increase in the PTB risk for an increase in PM_10_ concentrations among babies born in low-income groups [27] and Carbajal-Arroyo et al. in 2011 revealed a significant increase in the risk of all-cause mortality only among infants with low and medium SES [31]. These social inequalities in air pollution exposure of pregnant women and newborns are a public health issues. Additional studies are needed in Europe to improve our level of understanding concerning the underlying mechanisms explaining the existence of environmental inequality and to tackle this public health issues [32].

In epidemiological studies, quantifying the strength of the association between risk factors and health outcomes constitute pivotal information to document causality. However, these measures provide limited guidance for effective policies aimed at improving population health and reducing health inequalities. Spatial approaches may bring, in complement, useful information to help policymakers to elaborate on the choice of intervention. 

To our knowledge, few epidemiological studies investigated the spatial distribution of PTB. For instance, in Philadelphia, using a descriptive geographic-spatial approach conducted at census tract level, Boch et al. investigated the geographical patterns of the prevalence of PTB and examined its relationships with race, poverty, crime, and natality [33]. Today, the use of geographical information system for mapping adverse birth outcomes and maternal addresses, while more and more popular, is not sufficient to highlight areas that exhibit a higher risk. Additional spatial analyses are required to explore the spatial pattern of adverse birth outcomes and the spatial implication of neighborhood characteristics that may explain it. In Worcester, Ogneva-Himmelberger et al. in 2015 studied the spatial distribution of preterm births by racial groups to identify spatial clusters using mother’s residence address such as point location. Using two different spatial clustering methods, they analyzed associations between PTB and neighborhood characteristics including distance to major roads, exposure to hazardous air pollutants from stationary sources, access to vendors of healthy food, and access to green space and parks [34]. 

To our knowledge, no study has investigated the geographical distribution of PTB and its spatial association with the level of deprivation and the concentrations of air pollution measured at a small spatial scale. Indeed, to assess spatial patterns of health outcomes and its risk factors, fine spatial scale has been recommended in order to increase the homogeneity of specified variables within each area (such socioeconomic characteristics in this present study) and maximized differences between areas [35,36]; it is particularly important, when the study area, as in Paris city, presents a high population density per km^2^ with contrasted socioeconomic profiles. In addition, investigations of the spatial distribution of health events and risk factors conducted at the state or county level may not provide useful results for development of local policies or local decisions aiming to tackle social and environmental inequalities [37]. Small- spatial scale analyses appear to be an appropriated statistical unit to identify areas of high risk of PTB for targeted interventions and for reduction of inequalities in PTB.

In our study, spatial approaches appear to be the most appropriated to examine the spatial distribution of health risk and neighborhood characteristics. Using Kulldorf methods, we sought to perform clustering analysis to map the spatial distribution of the relative risks and to investigate the spatial implication of neighborhood characteristics. Unlike more traditional epidemiological studies which implement logistic regression to estimate impact of air pollution on the risk of preterm birth, with our approach we aim to answer to the same objective with an additional constraint related to spatial distribution of the health event. For example, Sabel et al. revealed that the relative risk (RR) of the pneumonia and influenza cluster adjusted for age, sex and deprivation is 1.92 whereas, the relative risk for the age, sex, deprivation and air pollution adjusted cluster is 1.99, respectively. However, these two clusters were not located in the same part of the territory and include different numbers of Census Area Unit (CAUs) while the risks estimated were similar [38]. More recently, Kihal et al. in France, found that the RR of end-stage renal disease (ESRD) incidence adjusted for sex, age and rural typology was 1.5, whereas the RR adjusted for age, sex and socioeconomic deprivation was 1.44. However, even estimated RR were similar; the two clusters were located at different part of the region: the first in the South-western part and the second in the extremely western Bretagne) and contained also different numbers of census blocks [39].

In this context, the localization of small geographical areas that exhibit a high PTB risk and their fine description may facilitate actions closely targeted towards areas most at risk: it is precisely the objective of this study. This work is not intended to reveal any causal pathway between neighborhood characteristics and PTB risk, an objective that requires other study designs [40,41]. 

## 2. Materials

### 2.1. Study Area 

The study area is the city of Paris which counts about 2,250,000 inhabitants. We used the smallest census unit area whose aggregate data can be used on a routine basis: the Ilots Regroupés pour l’Information Statistique (IRIS: the French acronym for ‘blocks for incorporating statistical information’). The IRIs is a sub-municipal French census block defined by the National Institute of Statistics and Economic Studies (INSEE). This statistical unit averages 2000 inhabitants and is constructed to be as homogenous as possible in terms of socioeconomic and demographic characteristics and land use. Paris is subdivided into 992 census blocks with a mean population of about 2199 inhabitants and a mean area of 0.11 km^2^.

### 2.2. Health Data: Preterm Birth

The preterm birth case has be defined according to the definition of World Health Organization (WHO) [42,43]: it corresponds to a neonate born before 37 weeks of pregnancy (gestational age ≤36 weeks). The preterm birth cases were identified from the first birth certificate information registered over the period 2008–2011 by the Maternal and Child Care department of Paris (named PMI, for Protection Maternelle et Infantile). This certificate is completed by parents and health professional before exit of the maternity and then sent to the PMI unit of the department of residence. 

All the postal addresses of mothers’ residency were geocoded at the census block level. For confidential concerns, to be in agreement with the ethical authorization provided for this study, it was not possible to keep individual localization of the newborn. The number of cases was aggregated at census blocks level for the statistical analysis. 

### 2.3. Air pollution: Nitrogen Dioxide (NO_2_)

Annual NO_2_ concentrations were modelled from a grid of 25 × 25 m resolution throughout the study period (2008–2011) by the local air quality monitoring networks corresponding to the Ile de France region (AirParif: http://www.airparif.asso.fr/). The ESMERALDA inter-regional platform for air quality mapping and forecasting (www.esmeralda-web.fr) provided background pollution data, while the STREET dispersion model [44] was used for traffic-related pollution. 

AirParif used a deterministic model which integrates various input parameters including linear (main roads), surface (diffuse road sources, residential and tertiary emissions) or industrial point sources and meteorological data (temperature, wind speed and direction, relative humidity, barometric pressure). More than 200 points sources were selected from the regional emission inventory. Emissions for traffic roads were estimated using the regional traffic network and the COPERT III European database for the 2002–2006 period, and COPERT IV for the 2007–2012 period. Concerning meteorological data, the Mesoscale Meteorological model (MM5: www.mmm.ucar.edu/mm5) developed by the Division of the NCAR Earth System Laboratory (NESL) was used. The NO_2_ background concentrations were determined by combining monitored NO_2_ concentrations from monitoring stations and those modeled at a regional scale from the ESMERALDA. The NO_2_ road traffic concentrations estimated from the STREET software model were added to NO_2_ background concentrations. Air pollutant concentrations were then aggregated at census block scale in order to obtain annual mean NO_2_ concentration for each census block (for more detail see Kihal-Talantikite et al. [45]; Deguen et al. [46]) over the study period. 

NO_2_ pollutant was chosen for several reasons: while data of PM_10_, PM_2.5_ and NO_2_ were available at the time of the study, we privileged the NO_2_ because this pollutant is recognized to be a good tracer of traffic and other combustion sources (major problems in Paris) [47]. It is also well known that the spatial variability of NO_2_ concentrations is higher than that of particulate matter [48]; a crucial point especially for spatial analysis; previous studies already revealed that exposure to NO_2_ may be related to adverse birth outcomes [11,12,49,50,51]. NO_2_ has been shown to be the best available indicators of local traffic emissions [48]. Finally, the correlation coefficients between NO_2_ and, PM10 and PM_2.5_ are very high: *r* = 0.95 and 0.93, respectively (see Figure S1 and Table S1 in Supplementary Materials).

### 2.4. Socioeconomic Deprivation Index

To characterize the level of socioeconomic deprivation at the census block scale, an index was created (details in another study [52]). The socioeconomic and demographic variables were provided by the 2010 national census at census block level. Briefly, a principal component analysis was used to select 15 variables out of 41 initial socioeconomic and demographic variables. Previous ecological studies have demonstrated this index’s ability to capture environment-related socio-spatial inequalities in France [53,54,55].

## 3. Methods

### 3.1. Spatial Methodology

To investigate the spatial distribution of PTB risk at census block level in Paris, we used a spatial scan statistic approach implemented in the SaTScan software [56]. 

The null hypothesis (H0) tests whether the risk of PTB is equi-distributed throughout the study area. The alternative hypothesis (H1) tests if there is an elevated PTB risk within the cluster in comparison with census blocks outside the cluster. 

In our study, the Poisson probability model implemented in the SaTScan software [56] was chosen as *cluster analysis method*. The number of PTB cases (a rare event) in each census block is assumed to follow a Poisson distribution. The input data for the Poisson model are the cases (PTB) and the population at risk (all birth) to determine if there is significant spatial clustering of the cases. 

We therefore compute a relative risk (RR) in each census block weighted by the population at risk count in each census block. The RR is estimated as the observed divided by the expected cases within the cluster divided by the observed divided by the expected cases outside the cluster (Equation (1)):

(1) RR=cE[c](C−c)(E[C]−E[c]=c/E[c](C−c)/(C−E[c])

where *c* is the number of observed PTB cases within the cluster and *C* is the total number of PTB cases in the data set. Note that since the analysis is conditioned on the total number of cases observed, *E*[*C*] = *C*.

The procedure to identify the most likely cluster is structured as follow. First, a circle of radius, varying from zero up to 50% of the population size [57], is placed at the centroid of every census blocks. Second, the circle moves across the study area to compare the PTB rate within the circle with what would be expected under a random distribution. Therefore, an infinite number of circles were created around each centroid, with the radius anywhere from zero up to a maximum so that at most 50 percent of the population is included. 

The scan statistic approach is likelihood based. The most likely cluster can be selected and tested for statistical significance. The likelihood function for the Poisson model is detailed in Equation (2):

(2) $${\left(\frac{c}{E\left(c\right)}\right)}^{c}{\left(\frac{C-c}{C-E\left(c\right)}\right)}^{C-c}I\;()$$

where *C* is the total number of PTB cases, c is the observed number of PTB cases within the window and *E*[*c*] is the covariate adjusted expected number of PTB cases within the window under the null hypothesis. Note that since the analysis is conditioned on the total number of cases observed, *C*-*E*[*c*] is the expected number of cases outside the window. *I* () is an indicator function. 

The identification of the most-likely clusters is based on a likelihood ratio test [58] with an associated *p*-value obtained using Monte Carlo replications [59]. The number of Monte Carlo replications was set to 999 to ensure adequate power for defining clusters and considered a 0.05 level of significance (*p* value derived from 999 replications).

### 3.2. Analytical Strategy and Results Interpretation

- When a significant most-likely cluster (with *p* < 0.05) was detected, the next step consist in taking into account the neighborhood characteristics to see whether or not the significant cluster can be explained by them. Spatial analyses were structured in four successive steps: A crude (unadjusted) analysis, to identify and localize the most-likely cluster of high risk of PTB.
- An adjusted analysis for NO_2_ concentrations
- An adjusted analysis for socioeconomic deprivation index
- One final adjusted analysis for air pollution and socioeconomic deprivation index including interaction between the two variables.

To incorporate covariates in the model, we categorized NO_2_ concentrations and socioeconomic deprivation index into five groups according to the quintile of their distribution. Because the SaTScan software does not allow for an interaction term to be accommodated in the model, we created several dummy variables combining the socioeconomic deprivation and the air pollution categories.

At the first step, a statistically significant test means that the risk of PTB is not randomly distributed in the city of Paris: a cluster of census blocks presents a significant increase in PTB risk in comparison with census blocks located outside the cluster [59].

For the three others steps, when the models are adjusted on one or more co-variables, according to the Kulldorff studies [57], several statistical criteria were used to test the H_0_ hypothesis: the cluster’s localization (the shift or the disappearance of the cluster, or no changes), the level of statistical significance of the cluster and the likelihood ratio value of each model. 

According to these criteria, there are three possible results: If, after adjustment, the most likely cluster remains in the same location, (whether or not this cluster is significant) and its likelihood ratio decreases, then it means that the variable(s) incorporated in the model explain partially the excess risk [56]; If the most likely cluster shifts (the centroid of the cluster changes), this suggests that the covariate(s) in the model explain the cluster’s excess risk [56] allowing the identification of second cluster. Finally, if the most likely cluster disappears totally, it means that the adjusted PTB risk is now randomly distributed over the study area. To map and visualize the spatial location of the statistically significant most likely clusters, we used ArcGis software (ESRI, Meudon, France).

## 4. Results

### 4.1. Description of the Population

**Figure 1:**
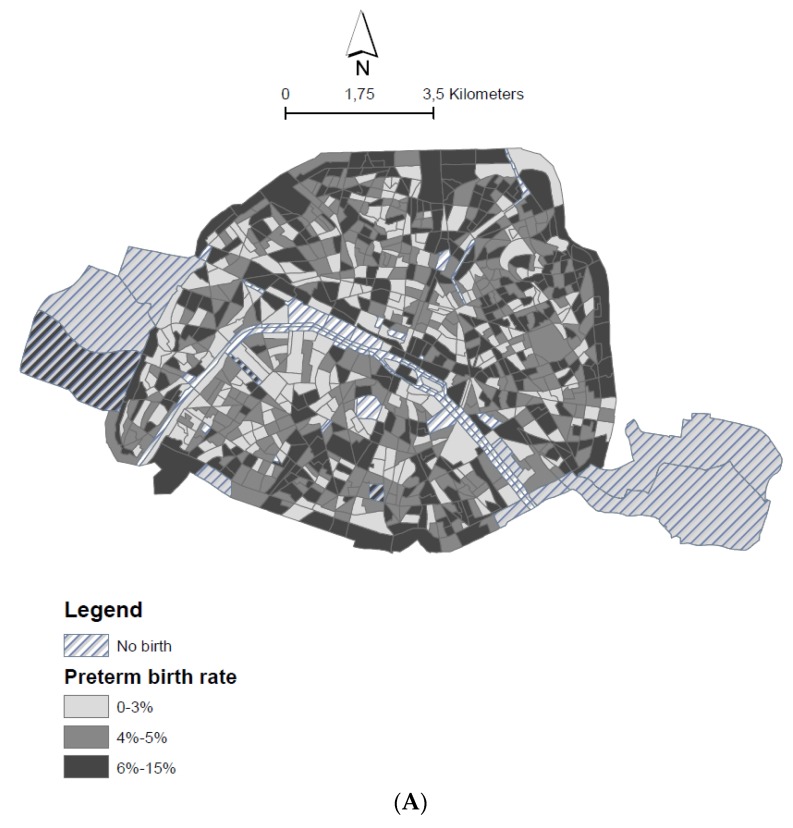

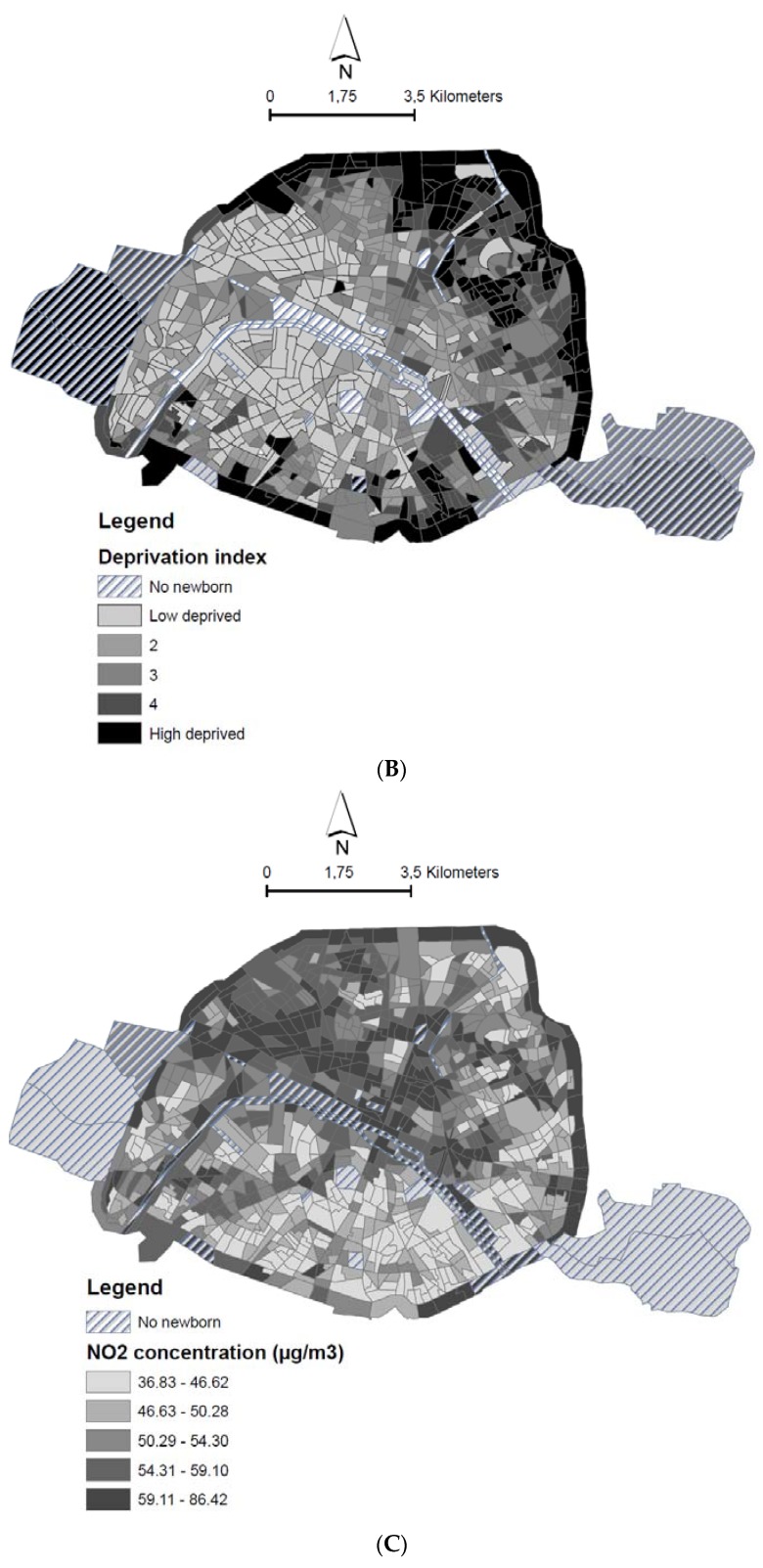
(**A**): Spatial distribution of crude preterm birth rate in census block areas within Paris; (**B**): Spatial distribution of socio-economic deprivation index in census block areas within Paris; (**C**): Spatial distribution of NO_2_ average concentrations from 2008 to 2011 in census block areas within Paris.

A total of 115,112 births were recorded during the study period 2008–2011. After exclusion of all birth with unknown birth weight, gestational age, with birth weights less or 500 g, we counted 110,746 singleton births (about 3.8% of the total births were excluded). When, we excluded also newborn without address (about 4.9%); the total singleton births include in the study is 105,346. Among them, 4871 births occurred before 37weeks of pregnancy. The rate of PTB in Paris was 4.6% overall during the study period (2008–2011). Figure 1A shows that low PTB rates, below 3%, are concentrated in the west-central part of Paris while in the north eastern areas, the PTB rate reaches 6 −15%. The geographical pattern of socioeconomic deprivation index is readily observed: the wealthiest census blocks are located in the western part of Paris while the most socioeconomically deprived neighborhoods are located in the northeast and along the perimeter (much trafficked highway) of Paris (Figure 1B). During study period, the PTB rate among women living in deprived census blocks is 4.9% (*n* = 241) compared to 3.18% (*n* = 155) among women living in less deprived census blocks (Significant Kruskal-Wallis test: *p*-value < 0.0001). 

All the census blocks have an annual average concentration of NO_2_ over the study period 2008–2011 higher than the European limit fixed to 40 μg/m^3^. The spatial distribution of the NO_2_ concentrations reveals a clear gradient from the north-western part of the city (the highest concentrations level >55.8 μg/m^3^) to the south-east part (the lowest concentrations level <50.6 μg/m^3^) (Figure 1C).

### 4.2. Neighborhood Socio-Economic Deprivation, NO_2_ Ambient Air Concentrations and Spatial Distribution of PTB in Paris

**Figure 2:**
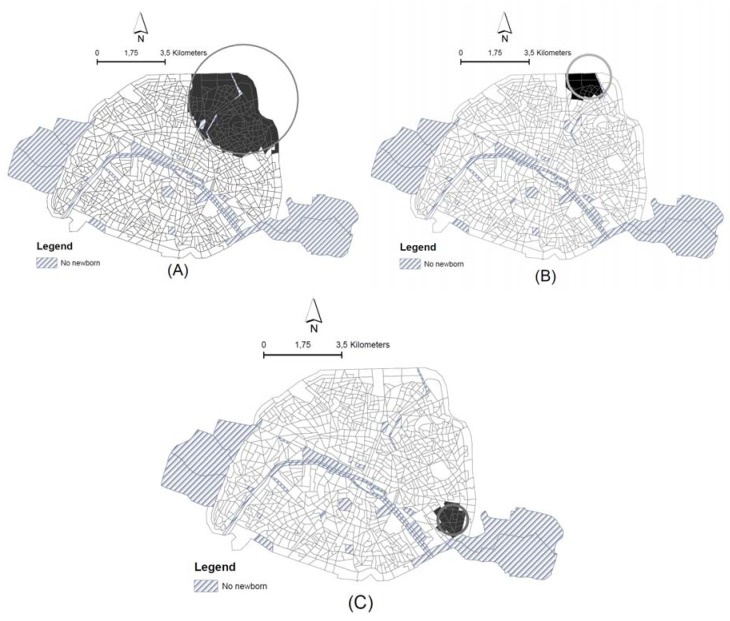
Spatial relocation of the most likely cluster of unadjusted PTB risk (**A**); after adjustment for NO_2_ exposure (**B**); after adjustment for NO_2_ exposure and socio-economic level (**C**). Legend: the dark area represents the census blocks included in the most likely cluster of high risk of PTB.

Figure 2 highlights the census blocks including in the most likely clusters of high risk of PTB, their location and spatial shift of centroids from unadjusted clusters to covariate-adjusted clusters. Table 1 summarizes the results of the spatial analyses: the most likely clusters, the number of census blocks, radius and relative risks (RR, the ratio of the observed- to-expected number in each census blocks estimated by SaTScan) for each cluster.

Unadjusted analysis (Figure 2A) reveals that the most likely cluster is located in the northeast part of Paris. Within the cluster, the risk of PTB is 1.15 times greater than in the rest of the study area (*p*-value < 0.06; Table 1). A total of 169 census blocks composes this most likely cluster, corresponding to about 25,503 inhabitants. The secondary cluster detected is not statistically significant (*p*-value = 0.89).

After adjustment for NO_2_ concentrations (Figure 2B), the most likely significant cluster is reduced (the radius decreases) and hosts 17 census blocks and 2,814 inhabitants. The risk of PTB increases in comparison with the crude estimate (RR = 1.40, *p*-value = 0.08). The centroid of the cluster shifts and the likelihood ratio slightly decreases from 9.23 (crude model) to 8.84 (adjusted model on air pollution) (Table 1), which suggests that the spatial distribution of NO_2_ concentrations partially explain the excess risk of PTB observed in the unadjusted analysis. 

After adjustment for socio-economic deprivation (Figure 2C), the most likely significant cluster shifts in South-Eastern Paris and the radius substantially decreases in size as well as the likelihood ratio (from 9.23 in the unadjusted model to 5.42) (Table 1). The remaining excess risk becomes not significant (RR = 1.34, *p*-value = 0.8). This indicates that socioeconomic deprivation explains a great part of the excess risk of PTB observed in the unadjusted analysis.

After joint adjustment for socioeconomic deprivation index and NO_2_ concentrations: the most likely cluster totally disappears. The likelihood ratio falls from 9.23 to 4.76; we also observed a likelihood ratio decrease when comparing with the model adjusted for socioeconomic deprivation index only (Table 1). The most likely cluster is not significant and located in the same zone in South-Eastern Paris (RR = 1.32; *p*-value = 0.97). This result indicates that the excess risk of PTB detected from the unadjusted analysis is entirely explained, by the spatial distribution of NO_2_ concentrations and socioeconomic deprivation.

In our study, the major finding is that while adjustment for socioeconomic deprivation level was the essential variable explaining the most likely cluster (as shown in Table 1), further adjustment for NO_2_ concentrations reduces the LLR to a larger degree than that obtained in the model with socioeconomic deprivation level alone or with the NO_2_ concentrations alone. 

## 5. Discussion

To our knowledge, such a work, exploring the spatial association of neighborhood characteristics on geographical variations of PTB at such a small-scale level had never been performed. For this reason it is difficult to compare our findings with others. Our study shows that neighborhood socioeconomic deprivation and average NO_2_ concentrations over years need to be considered in the interpretation of the spatial disparities in PTB in the city of Paris.

First, not surprisingly, NO_2_ concentrations only explained a very small part of the spatial variations of PTB across different census blocks. While several studies [12,18] have suggested that maternal exposure to ambient air pollutants (PM_10_, PM_2.5_, NO_2_) are associated with various birth outcome, the evidence regarding preterm birth is mixed and not conclusive. Some studies reported significant associations between exposure during pregnancy to NO_2_ and PTB [14,60,61,62,63], while others did not [20,64,65,66,67,68]. Recently, Estarlich et al. reported a suggestive association between residential exposure to NO_2_ during pregnancy and PTB among pregnant women who spent more time at home [69]. They found that exposure during the second trimester and during the whole pregnancy was associated with a higher risk of PTB. Johnson et al. in 2016, did not confirm the association between NO_2_ exposure and PTB in New York City. Using the proximity to traffic as a proxy for air pollution exposure, several studies show that the risk of preterm birth infants is significantly higher among mothers who live near freeways or roadways or to major roads [70,71,72,73,74,75].

Several biological pathways emerge from the literature to explain the potential impact of exposure to NO_2_ on PTB. Potential etiologic factors for PTB include inflammation, oxidative stress and cardiovascular alterations [76,77]. Some studies suggest that maternal exposures to NO_2_ can increase the risk of preterm delivery, via oxidative stress [78]. More recently, a second pathway through which NO_2_ could alter pregnancy outcomes was proposed. Some studies [61,73] suggest that traffic-related air pollution can related to some cause of PTB such as Preterm premature rupture of membranes (PROM).

Secondly, our findings revealed that the spatial distribution of neighborhood socioeconomic deprivation index explained a great part of spatial repartition of the excess risk of PTB observed in the crude analysis. This finding is coherent with previous works documenting the existence of a social gradient of adverse pregnancy outcome including PTB. Majority revealed an inverse association between PTB and various socioeconomic measures such as income [79,80,81,82,83], unemployment [84], composite socio-economic score including Towsend, carstairs or other socioeconomic deprivation index [85,86,87,88,89].

Recent literature review and meta-analysis concluded that living in a deprived neighborhood is associated with risk of preterm birth [40,41]. Vo et al. in 2014 found that odds ratios for preterm delivery significantly increased in the most deprived neighborhood quintile compared with the least deprived quintile (odds ratio 1.23, (95% CI:1.18–1.28)) [41]. Ncube et al. in 2016 estimated an excess risk of PTB equal to 27% (95%CI: 16%, 39%) among the most disadvantaged neighborhoods compared with least disadvantaged [40].

Many hypotheses have been formulated explaining the pathways through which socioeconomic status could be a potential risk factor of adverse pregnancy outcome including PTB:

(i) Psychological factors such as stressful life events or lack of social support, cohesive social networks and reciprocal exchanges between residents [90,91,92].
(ii) Unhealthy lifestyle featuring factors such as smoking or poor maternal nutrition and excess alcohol consumption especially around the time of conception [91,93,94,95,96,97].
(iii) Barriers and facilitating factors in access to healthcare such as availability of care, the ability to get to and pay for available care, or to seek and utilize available care [30,98].

The accumulation of these risk factors which is more common in deprived neighborhood [99], can contribute to maternal stress in turn can lead to higher levels of corticotropin-releasing hormone and cortisol which could trigger contractions and/or the premature rupture of the membrane resulting in PTB [100].

Finally, interestingly, our findings showed that the combination of socioeconomic deprivation level and NO_2_ concentrations, tacking account the interaction, explain a larger part of the excess risk of PTB estimated in the north-eastern Paris in comparison with analysis considering only socioeconomic deprivation level or NO_2_ concentrations (even if the contribution of air pollution is marginal compared to the one of socioeconomic deprivation index). These findings are coherent with previous epidemiological studies. For instance, in the U.S. State of Georgia, Hao et al. in 2015 [101] found that the strength of association between NO_2_ and PTB is higher for low education pregnant women. In California, Padula et al. 2014 confirmed a stronger association among pregnant women living in low socioeconomic status neighborhoods [60].

Two main hypotheses are more likely to explain the spatial implication of both NO_2_ exposure and socio-economic deprivation in geographical distribution of PTB.

(i) The first mechanisms—*vulnerability differential*—could explain the greater susceptibility to NO_2_ exposure of women living in the most deprived neighborhoods. Several studies demonstrated that people with a lower socio-economic status may be more vulnerable to the health effects of proximity to road, air pollution and noise exposure because they experience poorer health due to their economic and psychosocial conditions [15]. Living in communities with lower household income and education levels would also tend to increase vulnerability level to air pollution [102].
(ii) The second mechanism—*combined vulnerability differential with exposure differential—*may explain the greater susceptibility to NO_2_ exposure of women living in the deprived neighborhood. Although a majority of studies have found that people living in the most deprived neighborhoods may be more vulnerable to environmental nuisances, some authors have hypothesized that those living in middle deprived neighborhoods may have also a particular vulnerability. In this context, high NO_2_ exposure may act on this particular sensitive subpopulation, as an exacerbating factor, which, in combination with unfavorable living conditions, could generate greater health effects than in the rest of the population. The assumption of a synergy of differential exposure and vulnerability to explain our findings therefore seems highly probable.

Some research suggest that socioeconomic deprivation is spatially correlated with air pollution [103,104], and thus may have synergistic health effects through common biological pathways (e.g., chronic stress-induced inflammation, or dysregulation of immune and endocrine systems [105]). Clougherty et al. observed that a heightened susceptibility to pollution, associated with violence exposures or with fear thereof, may lead to synergistic health effects of social and physical environmental conditions. Bandoli et al. provide evidence of synergistic effects of air pollution and psychosocial stressors [106].

### Strengths and Limitations

One strength of this work is the use of small area-level analyses allowing a correct understanding of the geographic patterns of PTB. Moreover, this type of analysis is crucial for revealing local-level health inequalities that are often masked when analysis is produced at large spatial scales. 

Unlike geographical information system approach used to map and to visualize the spatial trends of PTB risk, in our study, we use a spatial clustering approach allowing us to identify areas of significantly elevated risk of PTB and to investigate spatial implications of adjustment for neighborhood characteristics. Another strength of our study is the databases used in our analysis to investigate PTB in France:

- *Health data:* the advantage of the data used in our study is the rate of completeness of the data which reach 93% on average and the large population size, resulting in a small variability of our estimates [107]. To our knowledge, this is the first French study investigate at fine spatial scale the birth certificates which list all birth in Paris during our study period.
- *Modeled air pollution data*: the air pollutant modeling procedure used provides unbiased estimates of exposure to ambient air pollution at census block level. This type of model was validated by Jerrett et al. who demonstrated its effectiveness and reliability [108].

However, the interpretation of our findings must also consider some weaknesses. Our approach, which uses ecological data, has several limitations. One is the absence of individual data such as maternal age, marital status and number of previous births. In addition, race/ethnic differences were not recorded in the first birth certificate because the French legislation prohibits the collection of any data based on race and ethnicity. Therefore, all statistical unit are considered equal. Data about the race/ethnicity were not available and were thus not included in our analysis. However, our study rests on a fine geographical resolution scale—census block—which has been designed by the Census bureau to be as homogeneous as possible in terms of population size and socio-economic and demographic characteristics. The level of homogeneity of the census blocks ensures the minimization of ecological bias, and the findings from this spatial analysis tend to be close to what could be observed at individual level [109,110]. Nonetheless, some degree of misclassification inevitably exists in individual characteristics and environmental exposures, and these could results in associations being biased towards the null. Another limitation is the absence of certain parental characteristics like lifestyle behaviors [92,95,111] including maternal nutritional deficits or status toxicants such as nicotine, cocaine or alcohol and access to healthcare [91,93,94,95,96,97,112]. Also, while socio-economic characteristics do not change rapidly over time, exposure to air pollution is highly variable and the present study considerer average NO_2_ concentrations over several years, the same value being assigned to all births that occurred in the same census block, irrespective of seasonality. From our data, the crude estimate of the PTB rate by season in Paris over the study period increase during the winter (rate of preterm equal 5.12%) while in summer the rate is 4.36%. A recent meta-analysis study [113] revealed that the pooled relative risks of preterm births increase during the winter months (maximum observed in January) and the beginning of summer (maximum observed in June). The air pollution concentrations follow similar temporal trend with the highest and lowest level in winter and in summer respectively. Indeed, the main emission sources of nitrogen oxides are road traffic (56%) and residential sector (18%) [114]. During summer, NO_2_ concentrations are lower, due to the slowdown of activities in the city and in particular the decrease of road traffic associated with the holiday period, and also in link with the chemistry of ozone formation. Due to the lack of data (we have not the daily concentrations of NO_2_ per census block), it was not possible to explore the air pollution PTB effect by season. This limitation is a common feature of ecological studies as the one we conducted. Epidemiological approaches that allow estimation of personal exposure information provide a complementary viewpoint, with their own limitations. 

Finally others characteristic such as green space could be associated with pregnancy outcome. In previous work we describe conceptual framework with 3 hypothetical pathways by which green spaces may have a beneficial effect on adverse pregnancy outcomes [55]. In addition, in recent study conducted in the same study area—city of Paris, we assessed the spatial variability of heat-wave-related mortality risk among elderly at the census block level and the most likely cluster for increased mortality risk is located in the same zone of cluster of high risk of PTB, in the East of Paris. In this study, we found that green space density had a protective effect [115]. In the future, It would be therefore interesting to collect all environmental exposures data from various sources, with negative health impacts (air, water and soil contamination, noise, etc.) or with positive effects (e.g., green space) and assess the effect of cumulative exposure on PTB risk using composite exposure index which performed in French [116]. 

## 6. Conclusions 

In a public health perspective, regarding maternal and child practice, individual-level interventions predominate. However, adverse birth outcomes result from a complex combination of individual determinants and behavior of the parents (more particularly the mother during pregnancy) and the characteristics of the place where people live requiring appropriate ecological approaches. 

Today, spatial approach constitutes a powerful tool to use in the context of the life course perspective of health, and more specifically in reproductive health [117]. A healthy pregnant woman is more likely to have a healthy newborn. In addition, neonates born in healthier place of residence in term of environmental exposure and, living and social conditions will tend to have better health trajectories throughout their life. The theoretical model suggests that adverse exposures (including characteristics of the place where people live) accumulate over time since the birth and will increase adverse outcomes during the adulthood period. In this context, this study is an attempt to fill the gap regarding a need for spatial approaches to support priority setting and guide policy makers in their choice of health interventions in general and on birth outcomes in particular. Our findings underscore the area with increased risk for preterm birth where local authorities should focus their resources and efforts to reduce health inequalities regarding birth outcomes. They highlight significant spatial implication of neighborhood characteristics including socioeconomic deprivation level and maternal exposure to ambient air NO_2_, and their combination, which could guide policymakers in choosing and developing the most appropriate and specific community-oriented interventions. We hope that in the future this kind of approach will be more often used in public health studies especially in life courses perspectives. 

## Figures and Tables

**Table 1:** Summary statistics of the most likely clusters of preterm birth risk and spatial relocation resulting from the adjustment analysis.

Analysis	Control Variables	Cluster Radius	No of Census Blocks/No. of Birth in the Cluster	No of Expected Cases	No. of Observed Cases	RR	LLr	Shift	*p*-Value
Unadjusted ^a^									
	No adjustment	2816.01	169/25,503	1179.94	1310	1.15	9.23	-	0.06
Adjusted ^b^									
	1 Annual concentration of NO_2_	1125.2	17/2814	130.84	181	1.40	8.84	Same zone	0.08
	SES ^c^ index	673.67	19/2396	104.95	140	1.34	5.42	Yes	0.81
	NO_2_ and SES level	673.67	19/2396	106.95	140	1.32	4.76	Yes	0.97

RR: relative risk; LLr: log likelihood ratio; ^a^ Unadjusted analysis, to identify and localize the most likely cluster(s) of high risk of PTB; ^b^ Adjusted analysis for (1) NO_2_ concentration, (2) socio-economic deprivation index, (3) NO_2_ concentration and socio-economic deprivation index; ^c^ Socio-economic deprivation index.

## Supplementary Materials

The following are available online at http://www.mdpi.com/1660-4601/15/9/1895/s1, Figure S1: Scatterplot matrix of NO_2_, PM_10_ and PM_2.5_, Table S1: Correlation Matrix between NO_2_, PM_10_ and PM_2.5_.

## References

[1] Terzidou V., Bennett P.R. (2002). Preterm labour. Curr. Opin. Obstet. Gynecol..

[2] Hack M., Klein N.K., Taylor H.G. (1995). Long-term developmental outcomes of low birth weight infants. Future Child.

[3] Lucas J.S., Inskip H.M., Godfrey K.M., Foreman C.T., Warner J.O., Gregson R.K., Clough J.B. (2004). Small size at birth and greater postnatal weight gain: relationships to diminished infant lung function. Am. J. Respir. Crit. Care Med..

[4] Barker D.J., Gluckman P.D., Godfrey K.M., Harding J.E., Owens J.A., Robinson J.S. (1993). Fetal nutrition and cardiovascular disease in adult life. Lancet.

[5] Mathews T.J., MacDorman M.F. (2008). Infant mortality statistics from the 2005 period linked birth/infant death data set. Natl. Vital Stat. Rep..

[6] Kramer M.S., Demissie K., Yang H., Platt R.W., Sauvé R., Liston R. (2000). The contribution of mild and moderate preterm birth to infant mortality. Fetal and Infant Health Study Group of the Canadian Perinatal Surveillance System. JAMA.

[7] Leem J.-H., Kaplan B.M., Shim Y.K., Pohl H.R., Gotway C.A., Bullard S.M., Rogers J.F., Smith M.M., Tylenda C.A. (2006). Exposures to Air Pollutants during Pregnancy and Preterm Delivery. Environ. Health Perspect..

[8] Mathews T.J., MacDorman M.F. (2007). Infant mortality statistics from the 2004 period linked birth/infant death data set. Natl. Vital Stat. Rep..

[9] Hutchinson E.A., De Luca C.R., Doyle L.W., Roberts G., Anderson P.J. (2013). Victorian Infant Collaborative Study Group School-age outcomes of extremely preterm or extremely low birth weight children. Pediatrics.

[10] Proietti E., Röösli M., Frey U., Latzin P. (2013). Air pollution during pregnancy and neonatal outcome: a review. J. Aerosol. Med. Pulm. Drug Deliv..

[11] Shah P.S., Balkhair T., Knowledge Synthesis Group on Determinants of Preterm/LBW births (2011). Air pollution and birth outcomes: A systematic review. Environ. Int..

[12] Stieb D.M., Chen L., Eshoul M., Judek S. (2012). Ambient air pollution, birth weight and preterm birth: A systematic review and meta-analysis. Environ. Res..

[13] Bonzini M., Carugno M., Grillo P., Mensi C., Bertazzi P.A., Pesatori A.C. (2010). Impact of ambient air pollution on birth outcomes: systematic review of the current evidences. Med. Lav..

[14] Wu J., Wilhelm M., Chung J., Ritz B. (2011). Comparing exposure assessment methods for traffic-related air pollution in an adverse pregnancy outcome study. Environ. Res..

[15] Miranda M.L., Edwards S.E., Chang H.H., Auten R.L. (2013). Proximity to roadways and pregnancy outcomes. J. Expo. Sci. Environ. Epidemiol..

[16] Johnson S., Bobb J.F., Ito K., Savitz D.A., Elston B., Shmool J.L.C., Dominici F., Ross Z., Clougherty J.E., Matte T. (2016). Ambient Fine Particulate Matter, Nitrogen Dioxide, and Preterm Birth in New York City. Environ. Health Perspect..

[17] Nieuwenhuijsen M.J., Dadvand P., Grellier J., Martinez D., Vrijheid M. (2013). Environmental risk factors of pregnancy outcomes: A summary of recent meta-analyses of epidemiological studies. Environ. Health.

[18] Bosetti C., Nieuwenhuijsen M.J., Gallus S., Cipriani S., La Vecchia C., Parazzini F. (2010). Ambient particulate matter and preterm birth or birth weight: A review of the literature. Arch. Toxicol..

[19] Chang H.H., Reich B.J., Miranda M.L. (2012). Time-to-event analysis of fine particle air pollution and preterm birth: Results from North Carolina, 2001-2005. Am. J. Epidemiol..

[20] Gehring U., Wijga A.H., Fischer P., de Jongste J.C., Kerkhof M., Koppelman G.H., Smit H.A., Brunekreef B. (2011). Traffic-related air pollution, preterm birth and term birth weight in the PIAMA birth cohort study. Environ. Res..

[21] Hansen C., Neller A., Williams G., Simpson R. (2006). Maternal exposure to low levels of ambient air pollution and preterm birth in Brisbane, Australia. BJOG.

[22] Ghio A.J., Carraway M.S., Madden M.C. (2012). Composition of air pollution particles and oxidative stress in cells, tissues, and living systems. J. Toxicol. Environ. Health B Crit. Rev..

[23] Dominici F., Sheppard L., Clyde M. (2003). Health Effects of Air Pollution: A Statistical Review. Int. Stat. Rev..

[24] Šrám R.J., Binková B., Dejmek J., Bobak M. (2005). Ambient Air Pollution and Pregnancy Outcomes: A Review of the Literature. Environ. Health Perspect..

[25] Strickland M.J., Klein M., Darrow L.A., Flanders W.D., Correa A., Marcus M., Tolbert P.E. (2009). The Issue of Confounding in Epidemiological Studies of Ambient Air Pollution and Pregnancy Outcomes. J. Epidemiol. Community Health.

[26] Sexton K., Gong H., Bailar J.C., Ford J.G., Gold D.R., Lambert W.E., Utell M.J. (1993). Air pollution health risks: Do class and race matter?. Toxicol. Ind. Health.

[27] Yi O., Kim H., Ha E. (2010). Does area level socioeconomic status modify the effects of PM_10_ on preterm delivery?. Environ. Res..

[28] Salihu H.M., Ghaji N., Mbah A.K., Alio A.P., August E.M., Boubakari I. (2012). Particulate pollutants and racial/ethnic disparity in feto-infant morbidity outcomes. Matern. Child Health J..

[29] Woodruff T.J., Parker J.D., Kyle A.D., Schoendorf K.C. (2003). Disparities in exposure to air pollution during pregnancy. Environ. Health Perspect..

[30] Ponce N.A., Hoggatt K.J., Wilhelm M., Ritz B. (2005). Preterm Birth: The Interaction of Traffic-related Air Pollution with Economic Hardship in Los Angeles Neighborhoods. Am. J. Epidemiol..

[31] Carbajal-Arroyo L., Miranda-Soberanis V., Medina-Ramón M., Rojas-Bracho L., Tzintzun G., Solís-Gutiérrez P., Méndez-Ramírez I., Hurtado-Díaz M., Schwartz J., Romieu I. (2011). Effect of PM(10) and O(3) on infant mortality among residents in the Mexico City Metropolitan Area: A case-crossover analysis, 1997–2005. J. Epidemiol. Community Health.

[32] OECD ENV/EPOC/WPNEP (2004). Rapport Environment and Distributional Issues: Analysis, Evidence and Policy Implications.

[33] Bloch J.R. (2011). Using Geographical Information Systems to Explore Disparities in Preterm Birth Rates among Foreign-born and U.S.-born Black Mothers. J. Obstet. Gynecol. Neonatal. Nurs..

[34] Ogneva-Himmelberger Y., Dahlberg T., Kelly K., Simas T.A.M. (2015). Using Geographic Information Science to Explore Associations between Air Pollution, Environmental Amenities, and Preterm Births. AIMS Public Health.

[35] Cockings S., Martin D. (2005). Zone design for environment and health studies using pre-aggregated data. Soc. Sci. Med..

[36] Haynes R., Daras K., Reading R., Jones A. (2007). Modifiable neighbourhood units, zone design and residents’ perceptions. Health Place.

[37] Insaf T.Z., Talbot T. (2016). Identifying areas at risk of low birth weight using spatial epidemiology: A small area surveillance study. Prev. Med..

[38] Sabel C.E., Wilson J.G., Kingham S., Tisch C., Epton M. (2007). Spatial implications of covariate adjustment on patterns of risk: Respiratory hospital admissions in Christchurch, New Zealand. Soc. Sci. Med..

[39] Kihal-Talantikite W., Deguen S., Padilla C., Siebert M., Couchoud C., Vigneau C., Bayat S. (2015). Spatial distribution of end-stage renal disease (ESRD) and social inequalities in mixed urban and rural areas: A study in the Bretagne administrative region of France. Clin. Kidney J..

[40] Ncube C.N., Enquobahrie D.A., Albert S.M., Herrick A.L., Burke J.G. (2016). Association of neighborhood context with offspring risk of preterm birth and low birthweight: A systematic review and meta-analysis of population-based studies. Soc. Sci. Med..

[41] Vos A.A., Posthumus A.G., Bonsel G.J., Steegers E.A.P., Denktaş S. (2014). Deprived neighborhoods and adverse perinatal outcome: A systematic review and meta-analysis. Acta Obstet. Gynecol. Scand..

[42] WHO Preterm Birth. http://www.who.int/news-room/fact-sheets/detail/preterm-birth.

[43] Quinn J.-A., Munoz F.M., Gonik B., Frau L., Cutland C., Mallett-Moore T., Kissou A., Wittke F., Das M., Nunes T. (2016). Preterm birth: Case definition & guidelines for data collection, analysis, and presentation of immunisation safety data. Vaccine.

[44] Vardoulakis S., Fisher B.E.A., Pericleous K., Gonzalez-Flesca N. (2003). Modelling air quality in street canyons: A review. Atmos. Environ..

[45] Wahida K.-T., Padilla C.M., Denis Z.-N., Olivier B., Géraldine L.N., Philippe Q., Séverine D. (2016). A Conceptual Framework for the Assessment of Cumulative Exposure to Air Pollution at a Fine Spatial Scale. Int. J. Environ. Res. Public Health.

[46] Deguen S., Petit C., Delbarre A., Kihal W., Padilla C., Benmarhnia T., Lapostolle A., Chauvin P., Zmirou-Navier D. (2015). Neighbourhood Characteristics and Long-Term Air Pollution Levels Modify the Association between the Short-Term Nitrogen Dioxide Concentrations and All-Cause Mortality in Paris. PLoS ONE.

[47] Padilla C.M., Kihal-Talantikit W., Vieira V.M., Deguen S. (2016). City-Specific Spatiotemporal Infant and Neonatal Mortality Clusters: Links with Socioeconomic and Air Pollution Spatial Patterns in France. Int. J. Environ. Res. Public Health.

[48] Levy I., Mihele C., Lu G., Narayan J., Brook J.R. (2014). Evaluating Multipollutant Exposure and Urban Air Quality: Pollutant Interrelationships, Neighborhood Variability, and Nitrogen Dioxide as a Proxy Pollutant. Environ. Health Perspect..

[49] Ritz B., Wilhelm M. (2008). Ambient Air Pollution and Adverse Birth Outcomes: Methodologic Issues in an Emerging Field. Basic Clin. Pharmacol. Toxicol..

[50] Brauer M., Lencar C., Tamburic L., Koehoorn M., Demers P., Karr C. (2008). A Cohort Study of Traffic-Related Air Pollution Impacts on Birth Outcomes. Environ. Health Perspect..

[51] Darrow L.A., Klein M., Flanders W.D., Waller L.A., Correa A., Marcus M., Mulholland J.A., Russell A.G., Tolbert P.E. (2009). Ambient air pollution and preterm birth: A time-series analysis. Epidemiology.

[52] Lalloué B., Monnez J.-M., Padilla C., Kihal W., Le Meur N., Zmirou-Navier D., Deguen S. (2013). A statistical procedure to create a neighborhood socioeconomic index for health inequalities analysis. Int. J. Equity Health.

[53] Kihal-Talantikite W., Padilla C.M., Lalloué B., Gelormini M., Zmirou-Navier D., Deguen S. (2013). Green space, social inequalities and neonatal mortality in France. BMC Pregnancy Childbirth.

[54] Kihal-Talantikite W., Padilla C.M., Lalloue B., Rougier C., Defrance J., Zmirou-Navier D., Deguen S. (2013). An exploratory spatial analysis to assess the relationship between deprivation, noise and infant mortality: An ecological study. Environ. Health.

[55] Padilla C.M., Deguen S., Lalloue B., Blanchard O., Beaugard C., Troude F., Navier D.Z., Vieira V.M. (2013). Cluster analysis of social and environment inequalities of infant mortality. A spatial study in small areas revealed by local disease mapping in France. Sci. Total Environ..

[56] Kulldorff M. (2005). SaTScan: Software for the Spatial, Temporal, and Space-Time Scan Statistics.

[57] Kulldorff M., Feuer E.J., Miller B.A., Freedman L.S. (1997). Breast cancer clusters in the northeast United States: A geographic analysis. Am. J. Epidemiol..

[58] Kulldorff M. (1997). A spatial scan statistic. Commun. Stat. Theory Methods.

[59] Dwass M. (1957). Modified Randomization Tests for Nonparametric Hypotheses. Ann. Math. Stat..

[60] Padula A.M., Mortimer K.M., Tager I.B., Hammond S.K., Lurmann F.W., Yang W., Stevenson D.K., Shaw G.M. (2014). Traffic-related air pollution and risk of preterm birth in the San Joaquin Valley of California. Ann. Epidemiol..

[61] Dadvand P., Basagaña X., Figueras F., Martinez D., Beelen R., Cirach M., de Nazelle A., Hoek G., Ostro B., Nieuwenhuijsen M.J. (2014). Air pollution and preterm premature rupture of membranes: A spatiotemporal analysis. Am. J. Epidemiol..

[62] Wilhelm M., Ghosh J.K., Su J., Cockburn M., Jerrett M., Ritz B. (2011). Traffic-related air toxics and preterm birth: A population-based case-control study in Los Angeles County, California. Environ. Health.

[63] Zhao Q., Liang Z., Tao S., Zhu J., Du Y. (2011). Effects of air pollution on neonatal prematurity in Guangzhou of China: A time-series study. Environ. Health.

[64] Hannam K., McNamee R., Baker P., Sibley C., Agius R. (2014). Air pollution exposure and adverse pregnancy outcomes in a large UK birth cohort: Use of a novel spatio-temporal modelling technique. Scand. J. Work Environ. Health.

[65] Gehring U., Tamburic L., Sbihi H., Davies H.W., Brauer M. (2014). Impact of noise and air pollution on pregnancy outcomes. Epidemiology.

[66] Schifano P., Lallo A., Asta F., De Sario M., Davoli M., Michelozzi P. (2013). Effect of ambient temperature and air pollutants on the risk of preterm birth, Rome 2001–2010. Environ. Int..

[67] Le H.Q., Batterman S.A., Wirth J.J., Wahl R.L., Hoggatt K.J., Sadeghnejad A., Hultin M.L., Depa M. (2012). Air pollutant exposure and preterm and term small-for-gestational-age births in Detroit, Michigan: Long-term trends and associations. Environ. Int..

[68] Van den Hooven E.H., Pierik F.H., de Kluizenaar Y., Willemsen S.P., Hofman A., van Ratingen S.W., Zandveld P.Y.J., Mackenbach J.P., Steegers E.A.P., Miedema H.M.E. (2012). Air pollution exposure during pregnancy, ultrasound measures of fetal growth, and adverse birth outcomes: A prospective cohort study. Environ. Health Perspect..

[69] Estarlich M., Ballester F., Davdand P., Llop S., Esplugues A., Fernández-Somoano A., Lertxundi A., Guxens M., Basterrechea M., Tardón A. (2016). Exposure to ambient air pollution during pregnancy and preterm birth: A Spanish multicenter birth cohort study. Environ. Res..

[70] Généreux M., Auger N., Goneau M., Daniel M. (2008). Neighbourhood socioeconomic status, maternal education and adverse birth outcomes among mothers living near highways. J. Epidemiol. Community Health.

[71] Yorifuji T., Naruse H., Kashima S., Ohki S., Murakoshi T., Takao S., Tsuda T., Doi H. (2011). Residential proximity to major roads and preterm births. Epidemiology.

[72] Yorifuji T., Naruse H., Kashima S., Takao S., Murakoshi T., Doi H., Kawachi I. (2013). Residential proximity to major roads and adverse birth outcomes: A hospital-based study. Environ. Health.

[73] Yorifuji T., Naruse H., Kashima S., Murakoshi T., Doi H. (2015). Residential proximity to major roads and obstetrical complications. Sci. Total Environ..

[74] Yang C.-Y., Chang C.-C., Chuang H.-Y., Ho C.-K., Wu T.-N., Tsai S.-S. (2003). Evidence for increased risks of preterm delivery in a population residing near a freeway in Taiwan. Arch. Environ. Health.

[75] Wilhelm M., Ritz B. (2003). Residential proximity to traffic and adverse birth outcomes in Los Angeles county, California, 1994–1996. Environ. Health Perspect..

[76] Vadillo-Ortega F., Osornio-Vargas A., Buxton M.A., Sánchez B.N., Rojas-Bracho L., Viveros-Alcaráz M., Castillo-Castrejón M., Beltrán-Montoya J., Brown D.G., O’Neill M.S. (2014). Air pollution, inflammation and preterm birth: A potential mechanistic link. Med. Hypotheses.

[77] Sapkota A., Chelikowsky A.P., Nachman K.E., Cohen A.J., Ritz B. (2012). Exposure to particulate matter and adverse birth outcomes: A comprehensive review and meta-analysis. Air Qual. Atmos. Health.

[78] Longini M., Perrone S., Vezzosi P., Marzocchi B., Kenanidis A., Centini G., Rosignoli L., Buonocore G. (2007). Association between oxidative stress in pregnancy and preterm premature rupture of membranes. Clin. Biochem..

[79] Luo Z.-C., Wilkins R., Kramer M.S., Fetal and Infant Health Study Group of the Canadian Perinatal Surveillance System (2006). Effect of neighbourhood income and maternal education on birth outcomes: A population-based study. CMAJ.

[80] Luo Z.-C., Kierans W.J., Wilkins R., Liston R.M., Mohamed J., Kramer M.S., British Columbia Vital Statistics Agency (2004). Disparities in birth outcomes by neighborhood income: Temporal trends in rural and urban areas, British Columbia. Epidemiology.

[81] Agyemang C., Vrijkotte T.G.M., Droomers M., van der Wal M.F., Bonsel G.J., Stronks K. (2009). The effect of neighbourhood income and deprivation on pregnancy outcomes in Amsterdam, The Netherlands. J. Epidemiol. Community Health.

[82] Liu N., Wen S.W., Katherine W., Bottomley J., Yang Q., Walker M.C. (2010). Neighbourhood family income and adverse birth outcomes among singleton deliveries. J. Obstet. Gynaecol. Can..

[83] Urquia M.L., Frank J.W., Glazier R.H., Moineddin R. (2007). Birth outcomes by neighbourhood income and recent immigration in Toronto. Health Rep..

[84] Garcia-Subirats I., Pérez G., Rodríguez-Sanz M., Ruiz-Muñoz D., Muñoz D.R., Salvador J. (2012). Neighborhood inequalities in adverse pregnancy outcomes in an urban setting in Spain: A multilevel approach. J. Urban Health.

[85] Janghorbani M., Stenhouse E., Millward A., Jones R.B. (2006). Neighborhood deprivation and preterm birth in Plymouth, UK. J. Matern. Fetal. Neonatal. Med..

[86] Gray R., Bonellie S.R., Chalmers J., Greer I., Jarvis S., Williams C. (2008). Social inequalities in preterm birth in Scotland 1980–2003: Findings from an area-based measure of deprivation. BJOG.

[87] Smith L.K., Draper E.S., Manktelow B.N., Field D.J. (2009). Socioeconomic inequalities in survival and provision of neonatal care: Population based study of very preterm infants. BMJ.

[88] Janevic T., Stein C.R., Savitz D.A., Kaufman J.S., Mason S.M., Herring A.H. (2010). Neighborhood deprivation and adverse birth outcomes among diverse ethnic groups. Ann. Epidemiol..

[89] Taylor-Robinson D., Agarwal U., Diggle P.J., Platt M.J., Yoxall B., Alfirevic Z. (2011). Quantifying the impact of deprivation on preterm births: A retrospective cohort study. PLoS ONE.

[90] Buka S.L., Brennan R.T., Rich-Edwards J.W., Raudenbush S.W., Earls F. (2003). Neighborhood support and the birth weight of urban infants. Am. J. Epidemiol..

[91] Schempf A., Strobino D., O’Campo P. (2009). Neighborhood Effects on Birthweight: An Exploration of Psychosocial and Behavioral Pathways in Baltimore, 1995–1996. Soc. Sci. Med..

[92] Nkansah-Amankra S., Luchok K.J., Hussey J.R., Watkins K., Liu X. (2010). Effects of maternal stress on low birth weight and preterm birth outcomes across neighborhoods of South Carolina, 2000–2003. Matern. Child Health J..

[93] Mitchell E.A., Robinson E., Clark P.M., Becroft D.M.O., Glavish N., Pattison N.S., Pryor J.E., Thompson J.M.D., Wild C.J. (2004). Maternal nutritional risk factors for small for gestational age babies in a developed country: A case-control study. Arch. Dis. Child. Fetal Neonatal Ed..

[94] Kozuki N., Lee A.C.C., Black R.E., Katz J. (2015). Nutritional and Reproductive Risk Factors for Small for Gestational Age and Preterm Births. Nestle Nutr. Inst. Works. Ser..

[95] Patra J., Bakker R., Irving H., Jaddoe V.W.V., Malini S., Rehm J. (2011). Dose-response relationship between alcohol consumption before and during pregnancy and the risks of low birthweight, preterm birth and small for gestational age (SGA)—A systematic review and meta-analyses. BJOG.

[96] Northstone K., Emmett P., Rogers I. (2008). Dietary patterns in pregnancy and associations with socio-demographic and lifestyle factors. Eur. J. Clin. Nutr..

[97] O’Leary C.M., Nassar N., Kurinczuk J.J., Bower C. (2009). The effect of maternal alcohol consumption on fetal growth and preterm birth. BJOG.

[98] Hsieh V.C.-R., Shieh S.-H., Chen C.-Y., Liou S.-H., Hsiao Y.-C., Wu T.-N. (2015). Does Social Health Insurance Close the Gap: The Case of Socioeconomic Status and Preterm Low-Birth-Weight Survival. Asia Pac. J. Public Health.

[99] Timmermans S., Bonsel G.J., Steegers-Theunissen R.P.M., Mackenbach J.P., Steyerberg E.W., Raat H., Verbrugh H.A., Tiemeier H.W., Hofman A., Birnie E. (2011). Individual accumulation of heterogeneous risks explains perinatal inequalities within deprived neighbourhoods. Eur. J. Epidemiol..

[100] Hodgson E.J., Lockwood C.J. (2010). Preterm Birth: A Complex Disease. Preterm Birth.

[101] Hao H., Chang H.H., Holmes H.A., Mulholland J.A., Klein M., Darrow L.A., Strickland M.J. (2016). Air Pollution and Preterm Birth in the U.S. State of Georgia (2002–2006): Associations with Concentrations of 11 Ambient Air Pollutants Estimated by Combining Community Multiscale Air Quality Model (CMAQ) Simulations with Stationary Monitor Measurements. Environ. Health Perspect..

[102] Cakmak S., Hebbern C., Cakmak J.D., Vanos J. (2016). The modifying effect of socioeconomic status on the relationship between traffic, air pollution and respiratory health in elementary schoolchildren. J. Environ. Manag..

[103] Clark L.P., Millet D.B., Marshall J.D. (2014). National Patterns in Environmental Injustice and Inequality: Outdoor NO_2_ Air Pollution in the United States. PLoS ONE.

[104] Tian N., Xue J., Barzyk T.M. (2013). Evaluating socioeconomic and racial differences in traffic-related metrics in the United States using a GIS approach. J. Expo. Sci. Environ. Epidemiol..

[105] Clougherty J.E., Kubzansky L.D. (2009). A Framework for Examining Social Stress and Susceptibility to Air Pollution in Respiratory Health. Environ. Health Perspect..

[106] Bandoli G., von Ehrenstein O., Ghosh J.K., Ritz B. (2016). Synergistic effects of air pollution and psychosocial stressors on adolescent lung function. J. Allergy Clin. Immunol..

[107] Premiers Certificats de Santé des Enfants nés en 2010 et Domiciliés en Île-de-France—Observatoire Régional de Santé Île-de-France—15 rue Falguière 75015 Paris—Tel.: 01 77 49 78 60—ors-idf@ors-idf.org. http://www.erpurs.org/index.php/fr/publications/56-groupes-de-population/meres-et-enfants/87-premiers-certificats-de-sante-des-enfants-nes-en-2010-et-domicilies-en-ile-de-france.

[108] Jerrett M., Gale S., Kontgis C. (2010). Spatial modeling in environmental and public health research. Int. J. Environ. Res. Public Health.

[109] Greenland S. (2001). Ecologic versus individual-level sources of bias in ecologic estimates of contextual health effects. Int. J. Epidemiol..

[110] Krieger N., Williams D.R., Moss N.E. (1997). Measuring social class in US public health research: Concepts, methodologies, and guidelines. Annu. Rev. Public Health.

[111] Miranda M.L., Maxson P., Edwards S. (2009). Environmental contributions to disparities in pregnancy outcomes. Epidemiol. Rev..

[112] De Graaf J.P., Steegers E.A.P., Bonsel G.J. (2013). Inequalities in perinatal and maternal health. Curr. Opin. Obstet. Gynecol..

[113] Beltran A.J., Wu J., Laurent O. (2013). Associations of meteorology with adverse pregnancy outcomes: A systematic review of preeclampsia, preterm birth and birth weight. Int. J. Environ. Res. Public Health.

[114] Airparif Inventaire Régional des Emissions en Ile-de-France—Année de Référence 2012. https://www.airparif.asso.fr/_pdf/publications/inventaire-emissions-idf-2012-150121.pdf,%22.

[115] Benmarhnia T., Kihal-Talantikite W., Ragettli M.S., Deguen S. (2017). Small-area spatiotemporal analysis of heatwave impacts on elderly mortality in Paris: A cluster analysis approach. Sci. Total Environ..

[116] Lalloué B., Monnez J.-M., Padilla C., Kihal W., Zmirou-Navier D., Deguen S. (2015). Data analysis techniques: A tool for cumulative exposure assessment. J. Expo. Sci. Environ. Epidemiol..

[117] Burton-Jeangros C., Cullati S., Sacker A., Blane D. (2015). A Life Course Perspective on Health Trajectories and Transitions.

