# Supplementary material for: Using a Clustering Approach to Investigate Socio-Environmental Inequality in Preterm Birth—A Study Conducted at Fine Spatial Scale in Paris (France)
Source: Int J Environ Res Public Health. 2018 Aug 31;15(9):1895. doi: 10.3390/ijerph15091895 (PMC6163167; doi:10.3390/ijerph15091895)
Supplement: Supplementary file 1 [file ijerph-15-01895-s001.pdf]

Supplementary Material

Using a Clustering Approach to Investigate Socio-Environmental Inequality in Preterm Birth—A Study Conducted at Fine Spatial Scale in Paris (France)

Severine Deguen [1,2,*], Nina Ahlers [1], Morgane Gilles [1], Arlette Danzon [3], Marion Carayol [3], Denis Zmirou-Navier [1,4,5], Wahida Kihal-Talantikite [6]

1 School of Public Health (EHESP), 35043 Rennes, Cedex, France; Nina.Ahlers@ucsf.edu (N.A.); morgane.gilles@ehesp.fr (M.G.); denis.zmirou@ehesp.fr (D.Z.-N.)
2 Department of Social Epidemiology, Institut Pierre Louis d'Epidémiologie et de Santé Publique (UMRS 1136), Sorbonne Universités, UPMC Univ Paris 06, INSERM, post code Paris, France
3 Service de Protection Maternelle et Infantile, Direction des Familles et de la Petite Enfance, Mairie de Paris, 75196 Paris, France; arlette.danzon@paris.fr (A.D.); marion.carayol@paris.fr (M.C.)
4 School of medicine, Lorraine University, 54000 Nancy, France
5 Inserm, Irset (Institut de recherche en santé, environnement et travail) -UMR-S 1085, F-35000 Rennes, France
6 Laboratoire Image Ville Environnement, LIVE UMR 7362 CNRS, University of Strasbourg, Strasbourg 6700, France; wahida.kihal@live-cnrs.unistra.fr (W.K.-T.)
* Correspondence: severine.deguen@ehesp.fr; Tel.: +332-99-02-2805

Figure S1. Scatterplot matrix of NO_2, PM_{10} and $PM_{2.5}$.

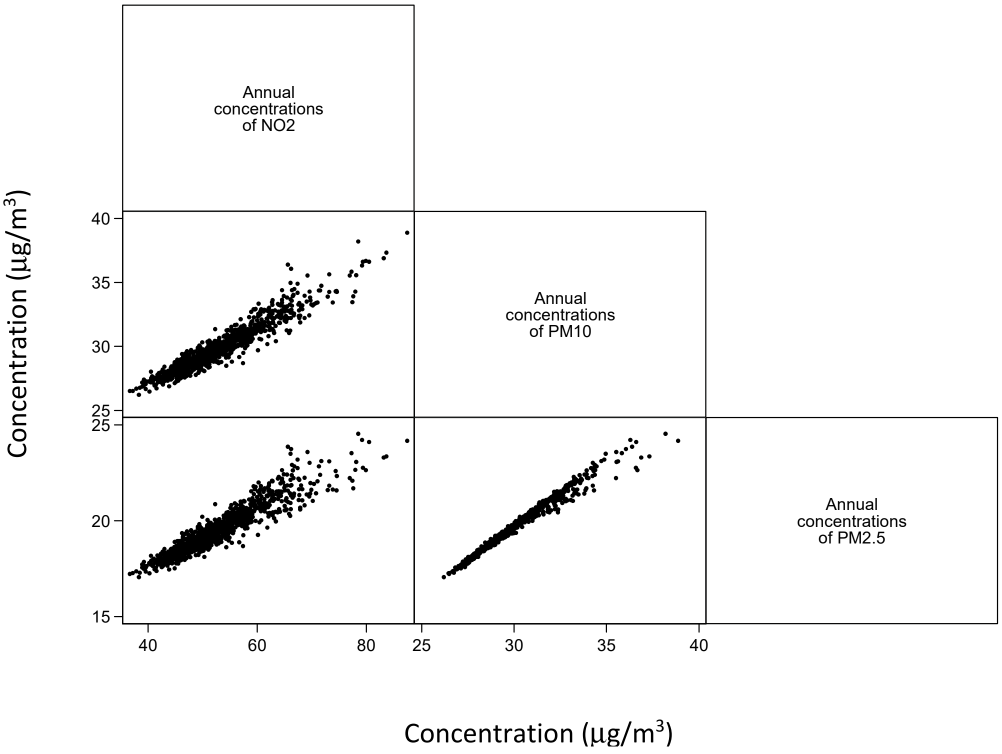

Table S1: Correlation Matrix between NO_2, PM_{10} and $PM_{2.5}$

Polluants	NO_2	PM_{10}	$PM_{2.5}$
NO_2	1		
PM_{10}	0.95	1	
$PM_{2.5}$	0.93	0.99	1
